# A Novel Blood Inflammatory Indicator for Predicting Deterioration Risk of Mild Traumatic Brain Injury

**DOI:** 10.3389/fnagi.2022.878484

**Published:** 2022-04-26

**Authors:** Xintong Ge, Luoyun Zhu, Meimei Li, Wenzhu Li, Fanglian Chen, Yongmei Li, Jianning Zhang, Ping Lei

**Affiliations:** ^1^Department of Geriatrics, Tianjin Medical University General Hospital, Tianjin, China; ^2^Tianjin Geriatrics Institute, Tianjin, China; ^3^Department of Medical Examination, Tianjin Medical University General Hospital, Tianjin, China; ^4^Key Laboratory of Immune Microenvironment and Disease, Department of Pathogen Biology, School of Basic Medical Sciences, Tianjin Medical University, Tianjin, China; ^5^Key Laboratory of Injuries, Variations and Regeneration of Nervous System, Key Laboratory of Post-trauma Neuro-repair and Regeneration in Central Nervous System, Tianjin Neurological Institute, Tianjin, China; ^6^Department of Neurosurgery, Tianjin Medical University General Hospital, Tianjin, China

**Keywords:** mild traumatic brain injury, risk factor, inflammation, neutrophil, lymphocyte, red cell distribution width, platelet

## Abstract

Mild traumatic brain injury (mTBI) has a relatively higher incidence in aging people due to walking problems. Cranial computed tomography and magnetic resonance imaging provide the standard diagnostic tool to identify intracranial complications in patients with mTBI. However, it is still necessary to further explore blood biomarkers for evaluating the deterioration risk at the early stage of mTBI to improve medical decision-making in the emergency department. The activation of the inflammatory response is one of the main pathological mechanisms leading to unfavorable outcomes of mTBI. As complete blood count (CBC) analysis is the most extensively used laboratory test in practice, we extracted clinical data of 994 patients with mTBI from two large clinical cohorts (MIMIC-IV and eICU-CRD) and selected inflammation-related indicators from CBC analysis to investigate their relationship with the deterioration after mTBI. The combinatorial indices neutrophil-to-lymphocyte ratio (NLR), red cell distribution width-to-platelet ratio (RPR), and NLR times RPR (NLTRP) were supposed to be potential risk predictors, and the data from the above cohorts were integratively analyzed using our previously reported method named MeDICS. We found that NLR, RPR, and NLTRP levels were higher among deteriorated patients than non-deteriorated patients with mTBI. Besides, high NLTRP was associated with increased deterioration risk, with the odds ratio increasing from NLTRP of 1–2 (2.69, 1.48–4.89) to > 2 (4.44, 1.51–13.08), using NLTRP of 0–1 as the reference. NLTRP had a moderately good prognostic performance with an area under the ROC curve of 0.7554 and a higher prediction value than both NLR and RPR, indicated by the integrated discrimination improvement index. The decision curve analysis also showed greater clinical benefits of NLTRP than NLR and RPR in a large range of threshold probabilities. Subgroup analysis further suggested that NLTRP is an independent risk factor for the deterioration after mTBI. In addition, *in vivo* experiments confirmed the association between NLTRP and neural/systemic inflammatory response after mTBI, which emphasized the importance of controlling inflammation in clinical treatment. Consequently, NLTRP is a promising biomarker for the deterioration risk of mTBI. It can be used in resource-limited settings, thus being proposed as a routinely available tool at all levels of the medical system.

## Introduction

Traumatic brain injury (TBI) ranges in severity, including the amount of brain damage and transient symptoms. Mild TBI (mTBI), the slightest neurotrauma, accounts for 80–90% of reported TBI cases and results in approximately 42 million emergency department visits worldwide each year (Gardner and Yaffe, 2015; Capizzi et al., 2020). Because of walking problems, mTBI has a relatively higher incidence in aging people. Most patients are characterized by a brief neurological impairment with no or only slight structural damage and a Glasgow Coma Scale (GCS) of 13–15 (Maas et al., 2017).

Cranial computed tomography (CT) is a standard diagnostic tool to identify intracranial complications of TBI. With its widespread application in the emergency department, doctors can quickly learn whether the individual has intracranial lesions, such as brain contusion, intracranial hemorrhage, subdural hematoma, and epidural hematoma, to propose the next-step medical suggestion for the patient—admit to the inpatient department, continue to stay in the emergency department for further diagnosis and treatment, or back home for rest and observation. Under normal circumstances, CT-negative patients or CT-positive patients with minor brain/cranial damage will choose to go home for observation or receive short-term treatments in the emergency and outpatient department. However, 11.7% of patients with mTBI may suffer from clinical deterioration with decreased GCS caused by secondary brain injury. Approximately 3.5% of patients with mTBI even need neurosurgical interventions (Marincowitz et al., 2018). Consequently, it is still necessary to further develop methods for evaluating the deterioration risk at the early stage of mTBI so that low-risk patients can be safely treated in the emergency department.

The use of blood biomarkers is an important supplementary tool for identifying patients with mTBI at deterioration risk. Recently, researchers have focused on several chemicals released from neural cells that may serve as biomarkers for neurological complications. S100B, a low-molecular-weight (21 kDa) protein that is mainly synthesized by astrocytes, could be rapidly released from damaged cells into circulation. Due to its excellent negative predictive value for mTBI, it has been regarded as an alternative choice for cranial CT scanning with appropriate evidence-based medicine (Undén et al., 2013). However, the determination of serum S100B needs a special measurement platform that ensures standardized and reproductive testing, and only three analyzers (Elecsys-Cobas, Roche; Liaison XL, Diasorin; and Vidas 3, bioMerieux) are in clinical use till date (Bouvier et al., 2016; Oris et al., 2019). Unfortunately, in most regions of the world, even in some high-level neurosurgical centers, the laboratory equipment are not supplied. For other potential biomarkers, including GFAP, UCH-L1, NFL, Tau, and some miRNAs, they have not been used in clinical work due to the lack of sufficient evidence from large-scale clinical trials and the compatible measurement platform in routine clinical practice. Taken together, there is an urgent need to find other blood biomarkers that are easily accessible and highly reliable to assist medical decision-making for patients with mTBI.

The activation of inflammation is one of the main pathological mechanisms leading to secondary brain injury and unfavorable outcomes in patients with mTBI (Le Bail et al., 2021). As complete blood count (CBC) analysis is the most extensively applied clinical laboratory test, we employed one of the largest reported mTBI cohorts in this study and screened the inflammation-related indicators from CBC analysis to investigate their relationship with the deterioration after mTBI. Specifically, the combinatorial indices neutrophil-to-lymphocyte ratio (NLR), RDW-to-platelet ratio (RPR), and NLR times RPR (NLTRP) were supposed to be potential risk predictors. The research findings are expected to provide solid evidence for the novel blood indicator as a routinely available biomarker at all levels of the medical system in predicting the outcome of mTBI.

## Materials and Methods

### Database Introduction

All data were extracted from the online international databases—Multiparameter Intelligent Monitoring in Intensive Care IV (MIMIC-IV, version 1.0) database and eICU Collaborative Research Database (eICU-CRD, version 2.0)—that are maintained by the Laboratory for Computational Physiology at the Massachusetts Institute of Technology (Cambridge, MA, United States). MIMIC-IV database was approved by the institutional review boards of the Massachusetts Institute of Technology and Beth Israel Deaconess Medical Center (Boston, MA, United States). It contains 523, 740 admissions at this medical center from 2008 to 2019, including a new ED module (not existing in MIMIC-III, the older version of the database) that contains data for patients while they are in the emergency department. EICU-CRD was released under the Health Insurance Portability and Accountability Act safe harbor provision, and the reidentification risk was certified as meeting safe harbor standards by Privacert (Cambridge, MA; Certification no. 1031219-2). It covers 200, 859 admissions in 2014 and 2015 from 208 hospitals across the United States. Besides, the source hospital of MIMIC-IV does not participate in the eICU-CRD program.

The data from the MIMIC-IV database and eICU-CRD are openly available. Data extraction was performed using Navicat Premium Version 12.1.11 (Preimumsoft™ CyberTech Ltd., Hongkong SAR, China).

### Study Population

Patients with a diagnosis of concussion in the MIMIC-IV database and intracranial injury in eICU-CRD were potentially eligible for inclusion in the mTBI cohort. If they had more than one hospitalization record, only those of the first hospital admission were kept. Patients were excluded if they met the following criteria: had no records of GCS within 24 h after hospital admission, had a record of GCS less than 13 during hospitalization, were younger than 18 years of age, had no binary gender, and/or had no records of whole blood RDW and platelet count within 24 h after hospital admission.

It should be mentioned that the lowest GCS of enrolled patients during hospitalization was not less than 13, so that the patients with moderate or severe TBI exacerbated by mTBI were excluded. Although these patients do not show severe symptoms in the acute stage of TBI, most of them can be observed with serious intracranial injuries by cranial CT and obvious changes in consciousness when the condition gets worse. Doctors can use the existing conventional examination methods to fully judge the condition and risks, thus making medical decisions on hospital admission and even neurosurgery. Therefore, these patients were not included in this study.

### Data Extraction

Structure Query Language was used to extract data from the two databases. The following information was extracted: age, gender, important past history and comorbidities, GCS records, neutrophil count, lymphocyte count, RDW, and platelet count within 24 h after hospital admission. The past history and comorbidities include arteriosclerotic heart disease (ASHD), chronic obstructive pulmonary disease (COPD), high blood pressure (HBP), liver failure or cirrhosis, renal failure or uremia, stroke, malignancy, and use of antiplatelet/anticoagulant/antithrombotic drugs. For patients with more than one record of the neutrophil count, lymphocyte count, RDW, or platelet count within 24 h after hospital admission, the average value was calculated, respectively, and NLR, RPR, and NLTRP were then figured out using these values. In addition, the outcome of the study was designed to be a deterioration in GCS, which was defined as a decline from a 14 or 15 scale at admission to a 13 scale (the lowest scale of mTBI) within 24 h after hospital admission.

To integrate the data from the MIMIC-IV database and eICU-CRD, we used our self-developed MeDICS (MIMIC-IV and eICU Database Integrated Cohort Study) method as previously reported (Ge et al., 2021). This method could be applied to establish diagnostic and prognostic models for various diseases, and its value in exploring the biomarker for acute TBI has been verified by a retrospective clinical study with the same research objective using an external cohort (Wang et al., 2021). Briefly, ICU-stay-ID or patient-unit-stay-ID was regarded as the unique ID for each patient in the cohort. Incompatible data, such as patient-health system-stay-ID, were excluded. Besides, as the two databases may have different definitions of the same diseases, the disease codes were unified by manual review. Furthermore, the same variables with inconsistent data types in the two databases, such as numbers and strings, were also unified.

### Management of Missing Data and Outliers

Variables with missing data are common in the MIMIC-IV database and eICU-CRD. As described in the “Study population” section, patients with missing records of GCS, neutrophil count, lymphocyte count, RDW, or platelet count within 24 h after hospital admission were excluded from the analysis. Variables with more than 20% missing values, such as patients’ height and weight, were also excluded. In addition, the NLTRP outliers with more than 3.00, the upper quartile plus 1.5-fold of interquartile range, were excluded as non-normally distributed outliers, along with their related data, including NLR and RPR.

### Animals and Grouping Methods

Adult male C57BL/6 mice (*n* = 72, aged 12 weeks, weighing 20–25 g) were purchased from the Chinese Academy of Military Science (Beijing, China). All experimental procedures were conducted in accordance with the EC Directive 86/609/EEC for animal experiments and were approved by Tianjin Medical University Animal Care and Use Committee (Grant No. IRB2021-DWFL-359). The mice were quarantined and housed for 2 weeks before being randomly divided into 4 groups (*n* = 6/group, 3 independent experiments): sham, TBI, TBI + SC75741, and TBI + MCC950. The treatment was administered at 1 h post-injury. Briefly, SC75741 (Selleckchem, Houston, TX, United States), the NF-κB selective inhibitor, was dosed in the mice at 15 mg/ml intraperitoneally. MCC950 (Selleckchem), the specific inhibitor of pyroptosis initiating receptor NLRP3, was applied to the mice at 10 mg/kg intraperitoneally.

### Mild Closed Head Injury Mouse Model

The mice were anesthetized with 4.6% isoflurane. A molded acrylic cast was designed to fix the head and provide a 3.0-mm space below for acceleration and deceleration beneath the point of impact, and the surgical tape was used to secure the mouse in a prone position. After shaving the head, a self-designed standard manufacturing concave metal disk was adhered to the skull immediately caudal to the bregma as a helmet. The impounder tip of the injury device (eCCI model 6.3, American Instruments, Richmond, VA, United States) was then extended to its full impact distance, positioned on the center of the disk surface, and reset to induce a closed head injury. The impact parameters were set as follows: 5.0 m/s velocity, 2.5 mm depth, and 200 ms dwell time. After the impact procedure, the mice were placed in a well-ventilated cage at 37^°^C until they regained consciousness. The sham-operated mice underwent the same procedures except for the impact (Ge et al., 2018).

### Complete Blood Count Analysis

The mice were anesthetized with 4.6% isoflurane by intraperitoneal injection at 6 h post-injury. Whole blood samples were then collected *via* cardiac puncture and were anticoagulated with heparin. To determine neutrophil count, lymphocyte count, RDW, and platelet count, the blood sample was sent for CBC analysis using Sysmex XN-2000 Automated Hematology Analyzer (Kobe, Hyogo, Japan).

### Enzyme Linked Immunosorbent Assay

Peripheral blood was drawn by cardiac puncture at 6 h post-injury for serum inflammatory indicator measurement. After that, the mice were sacrificed by transcardiac perfusion with PBS. The injured cerebral cortex was then dissected to collect brain extracts. The ELISA assay of the inflammatory mediators, including TNF-α, IL-1β, and IL-10, was determined by referring to the manufacturer’s instructions (Cat# MTA00B, MLB00C, M1000B; R&D, Minneapolis, MN, United States).

### Statistical Analysis

For the data collected from the MIMIC-IV database and eICU-CRD, continuous variables were expressed as mean ± *SD* and compared using Student’s *t*-test, the Wilcoxon rank-sum test, or the Kruskal–Wallis rank-sum test, as appropriate. Categorical data were expressed as numbers (percentages) and compared using the chi-square test.

The association between NLTRP and the deterioration risk was determined using the logistic regression model and presented as an odds ratio (OR) with a 95% confidence interval (CI). NLTRP values were divided into three groups of tertiles, with the first tertile (0–1) selected as the reference group. The Lowess Smoothing technique was used to explore the crude relationship between NLTRP and the deterioration risk. Multivariable logistic regression analyses were used to control confounders. Model 1 was adjusted for the confounders, namely, age and gender. Model 2 was adjusted for the confounders, namely, age, gender, history, and comorbidities, including ASHD, COPD, HBP, liver failure or cirrhosis, renal failure, or uremia, stroke, malignancy, and use of antiplatelet/anticoagulant/antithrombotic drugs. These confounders were selected based on their potential influences on NLTRP or the clinical course. Potential multicollinearity was tested using the variance inflation factor, with a value of ≥ 5 indicating the presence of multicollinearity. The receiver-operating characteristic (ROC) curve was depicted to show the outcome prediction performance and confirm the best cutoff value, and the integrated discrimination improvement (IDI) index was calculated to compare the predictive values of different indicators. Decision curve analysis (DCA) was further performed to evaluate the clinical benefits of various indicators. In addition, stratification in subgroup analysis was performed according to age, gender, history, and comorbidities to explore their possible interaction with NLTRP to determine the deterioration risk.

The data from the *in vivo* study were expressed as mean ± standard deviation, and the statistical comparisons were made using Student’s *t*-test or one-way ANOVA followed by the Tukey HSD *post-hoc* test, as appropriate.

All statistical analyses were performed using Stata/MP Version 14.0 (Stata Corp., College Station, TX, United States) and PASW Statistics Version 18.0 (IBM, Armonk, NY, United States). A two-tailed *p*-value of less than 0.05 was considered to be statistically significant.

## Results

### Study Population and Baseline Characteristics

**FIGURE 1:**
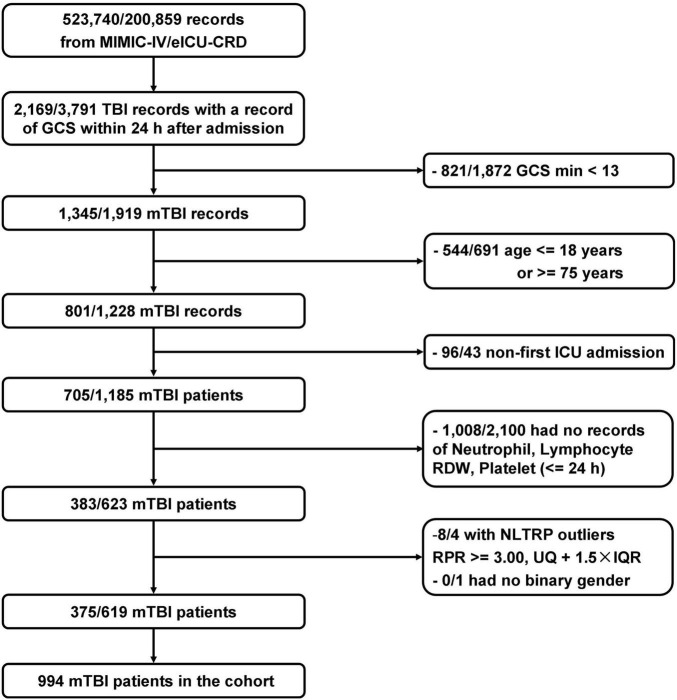
Flowchart of the study population. In total, 994 patients who met the selection criteria were enrolled. GCS, Glasgow Coma Scale; IQR, interquartile range; mTBI, mild traumatic brain injury; NLTRP, neutrophil-to-lymphocyte ratio times red cell distribution width-to-platelet ratio; RDW, red cell distribution width; UQ, upper tertile.

In total, 994 patients who met the selection criteria were enrolled in the cohort, which included 889 non-deteriorated subjects and 105 deteriorated subjects, establishing a deterioration rate of 10.6%. The detailed procedure for population selection is shown in Figure 1. Demographic characteristics of the cohort are presented in Table 1. The patients with mTBI in the cohort had an average age of 53.27 years, which was consistent with the recent report on the incidence of TBI in America (Peterson and Thomas, 2021). No difference in the deterioration risk was observed based on age and gender. Besides, important history and comorbidities during hospitalization, including ASHD, COPD, HBP, liver failure or cirrhosis, renal failure or uremia, stroke, malignancy, or use of antiplatelet/anticoagulant/antithrombotic drugs, also did not affect the deterioration after mTBI. For the indicators of the CBC analysis, neutrophil count, lymphocyte count, RDW, and platelet count did not show independent differences between non-deteriorated and deteriorated subjects. However, it was worth noting that the combinatorial indices NLR (*p* = 0.0017), RPR (*p* = 0.0011), and NLTRP (*p* < 0.001) were all lower for non-deteriorated subjects than deteriorated subjects.

**TABLE 1:** Baseline characteristics of the patients with mTBI.

Variables	Total (*n* = 994)	Non-deteriorated (*n* = 889)	Deteriorated (*n* = 105)	*p*-value
Age, Years	53.27 ± 16.27	53.06 ± 16.35	55.08 ± 15.53	0.115
Male, *n* (%)	665 (66.9)	592 (66.6)	73 (69.5)	0.541
**Past history and comorbidities, *n* (%)**				
ASHD				0.807
Yes	70 (7.0)	62 (7.0)	8 (7.6)	
No	924 (93.0)	827 (93.0)	97 (92.4)	
COPD				0.799
Yes	16 (1.6)	14 (1.6)	2 (2.0)	
No	978 (98.4)	875 (98.4)	103 (98.0)	
HBP				0.265
Yes	196 (19.7)	171 (19.2)	25 (23.8)	
No	798 (80.3)	718 (80.8)	80 (76.2)	
Liver failure or cirrhosis				0.635
Yes	22 (2.2)	19 (2.1)	3 (2.9)	
No	972 (97.8)	102 (97.9)	870 (97.1)	
Renal failure or uremia				0.695
Yes	49 (4.9)	43 (4.8)	6 (5.7)	
No	945 (95.1)	846 (95.2)	99 (94.3)	
Stroke				0.719
Yes	24 (2.4)	22 (2.2)	2 (1.9)	
No	970 (97.6)	867 (97.8)	103 (98.1)	
Malignancy				0.871
Yes	17 (1.7)	15 (1.7)	2 (1.9)	
No	977 (98.3)	874 (98.3)	103 (98.1)	
Antiplatelets/-coagulants/-thrombotics				0.749
Yes	64 (6.4)	58 (6.5)	6 (5.7)	
No	930 (93.6)	831 (93.5)	99 (94.3)	
**CBC analysis**				
Neutrophil, K/μL	245.37 ± 433.29	245.38 ± 436.14	245.26 ± 410.35	0.501
Lymphocyte, K/μL	43.92 ± 79.18	43.85 ± 76.57	44.45 ± 98.94	0.471
RDW,%	91.21 ± 84.00	91.46 ± 115.44	89.14 ± 120.58	0.577
Platelet, K/μL	220.55 ± 85.62	222.30 ± 82.44	205.71 ± 108.22	0.970
NLR	6.50 ± 5.48	6.33 ± 5.36	7.98 ± 6.21	0.0017**
RPR	0.076 ± 0.055	0.074 ± 0.047	0.096 ± 0.086	0.0011**
NLTRP	0.463 ± 0.421	0.438 ± 0.389	0.673 ± 0.485	< 0.001***

The data were expressed as mean ± SD or n (%). ***p < 0.001, **p < 0.01. ASHD, arteriosclerotic heart disease; CBC, complete blood count; COPD, chronic obstructive pulmonary disease; HBP, high blood pressure; NLR, neutrophil-to-lymphocyte ratio; RDW, red cell distribution width; RPR, red cell distribution width-to-platelet ratio; NLTRP, neutrophil-to-lymphocyte ratio times red cell distribution width-to-platelet ratio.

### High NLTRP Is Related to Increased Deterioration Risk of mTBI

**FIGURE 2:**
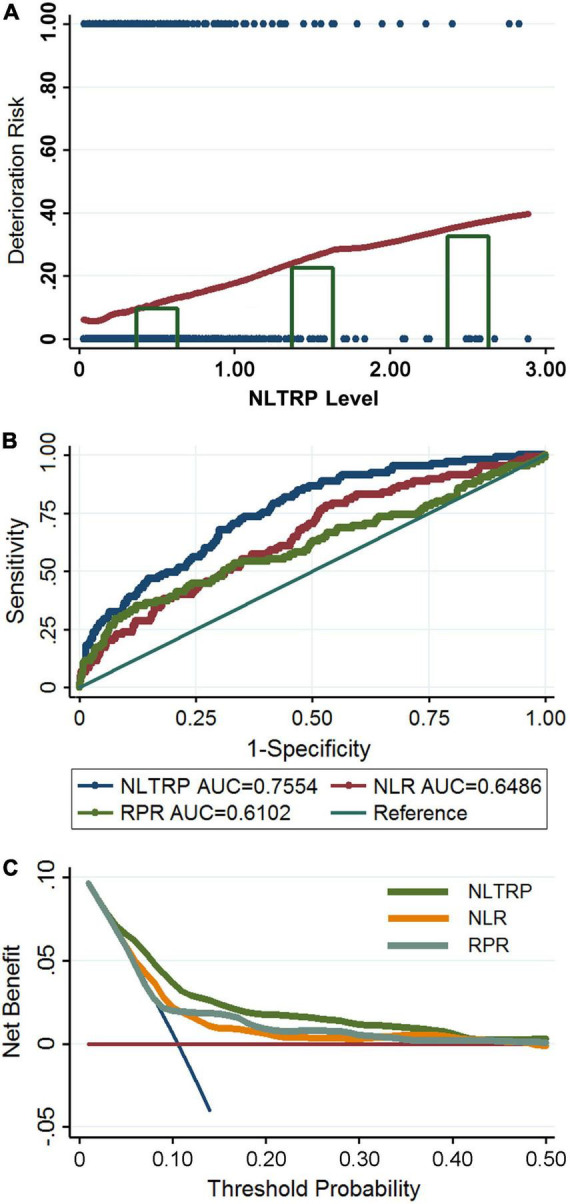
Association between NLTRP and the deterioration risk of patients with mTBI. **(A)** The Lowess Smoothing revealed an approximately linear relationship between NLTRP and the deterioration risk. The NLTRP values were divided into tertiles, and the third (>2) and the second tertiles (1–2) were associated with high deterioration risk, compared to the first (0–1) tertile. The blue dot represents each patient enrolled in the cohort. Individuals with a deterioration outcome were presented with the risk of 1, and those with a non-deterioration outcome were presented with a risk of 0. **(B)** The ROC curve for NLTRP, NLR, and RPR. **(C)** The DCA for NLTRP, NLR, and RPR. AUC, area under curve; DCA, decision curve analysis; ROC, receiver-operating characteristic; mTBI, mild traumatic brain injury; NLR, neutrophil-to-lymphocyte ratio; RPR, red cell distribution width-to-platelet ratio; NLTRP, neutrophil-to-lymphocyte ratio times red cell distribution width-to-platelet ratio.

The relationship between NLTRP and deterioration risk for patients with mTBI is shown in Figure 2A using the Lowess Smoothing technique, which yielded an approximately linear correlation. NLTRP values were divided into three groups of tertiles (NLTRP 1–2, *n* = 74; NLTRP > 2, *n* = 16), with the first tertile (NLTRP 0–1, *n* = 904) being selected as the reference for all comparisons in multivariable logistic regression models. As shown in Table 2, the ORs with 95% CI for the second, (1–2) and third (> 2) tertiles in the crude model were 2.69 (1.48–4.89) and 4.44 (1.51–13.08), respectively. Therefore, high NLTRP was associated with an increased deterioration risk of mTBI. Besides, a similar trend could be observed in Model 1 and Model 2, in which the confounders, including age, gender, history, and comorbidities, were adjusted. It also suggested that the second and third tertiles had successively higher ORs (95% CI) than the first tertile in all models. In addition, the mean-variance inflation factor was 1.33 and 1.26 for Model 1 and Model 2, indicating that no multicollinearity was existed.

**TABLE 2:** The ORs for all-cause deterioration of mTBI across groups of NLTRP.

NLTRP levels	OR	95% CI	*p*-value
**Crude**			
0–1	1		
1–2	2.69	1.48–4.89	0.001**
>2	4.44	1.51–13.08	0.007**
**Model 1**			
0–1	1		
1–2	2.60	1.43–4.74	0.002**
>2	4.27	1.45–12.64	0.009**
**Model 2**			
0–1	1		
1–2	2.59	1.42–4.73	0.002**
>2	4.43	1.48–13.25	0.008**

Multivariable logistic regression models were used to calculate ORs with 95% CI. Model 1 was adjusted for the confounders, namely, age and gender. Model 2 was adjusted for the confounders, namely, age, gender, past history, and comorbidities, including ASHD, COPD, HBP, liver failure or cirrhosis, renal failure or uremia, stroke, malignancy, and use of antiplatelet/anticoagulant/antithrombotic drugs. The mean-variance inflation factor was 1.33 and 1.26 for Model 1 and Model 2. **p < 0.01. ASHD, arteriosclerotic heart disease; CI, confidence interval; COPD, chronic obstructive pulmonary disease; HBP, high blood pressure; NLTRP, neutrophil-to-lymphocyte ratio times red cell distribution width-to-platelet ratio; OR, odds ratio.

The outcome prediction value of NLTRP was examined using the ROC curve. As shown in Figure 2B, its performance was moderately good for the deterioration risk (AUC = 0.7554) with a cutoff value (Youden’s index) of 0.0729, corresponding to a sensitivity of 81.90% and specificity of 56.13%. It is much higher than that of NLR (AUC = 0.6486) and RPR (AUC = 0.6102). IDI index also indicated NLTRP had a higher outcome prediction value than NLR (0.0879 ± 0.0196, *p* < 0.0001) and RPR (0.0546 ± 0.0150, *p* = 0.0003). In addition, the results of DCA for NLTRP, NLR, and RPR are shown in Figure 2C. It further suggests that the clinical benefits of NLTRP are greater than both NLR and RPR across a large range of threshold probabilities.

### Subgroup Analysis Confirms the Association Between NLTRP and Deterioration Risk of mTBI

As shown in Table 3, a subgroup analysis revealed the associations between NLTRP and the deterioration risk of patients with mTBI of different ages, gender, past history, and comorbidities, including ASHD, HBP, renal failure, or uremia, and use of antiplatelet/anticoagulant/antithrombotic drugs. As the number of patients with COPD, liver failure or cirrhosis, stroke, and malignancy was too small in the cohort (especially for the deteriorated subjects), their subgroup analysis could not be performed. After adjusting for covariates, the interactive effects could not be observed in all subgroups, suggesting that NLTRP is an independent risk factor for the deterioration after mTBI.

**TABLE 3:** Subgroup analysis of the associations between NLTRP and deterioration of mTBI.

Subgroups	ORs (95% CI)			*p* for interaction
	NLTRP 0–1	NLTRP 1–2	NLTRP > 2	
Age				0.055
18–45	1	3.54 (0.68–18.48)	U.C.	
45–60	1	2.60 (0.87–7.73)	8.15 (1.27–52.43)	
60–75	1	2.13 (0.90–5.07)	3.07 (0.55–17.11)	
Gender				0.152
Male	1	1.95 (0.78–4.87)	3.50 (1.47–8.30)	
Female	1	0.77 (0.42–1.42)	1.33 (0.76–2.33)	
ASHD				0.499
Yes	1	1.89 (0.06–24.82)	5.26 (1.26–48.46)	
No	1	2.71 (1.44–5.09)	3.91 (1.17–13.03)	
HBP				0.885
Yes	1	2.03 (0.59–7.05)	3.72 (0.30–45.91)	
No	1	2.93 (1.44–5.92)	4.27 (1.23–14.79)	
Renal failure or uremia				0.495
Yes	1	1.17 (0.43–9.16)	3.51 (0.04–33.8)	
No	1	2.36 (1.25–4.47)	4.21 (1.25–14.12)	
Antiplatelets/-coagulants/-thrombotics				0.299
Yes	1	2.77 (0.19–40.10)	U.C.	
No	1	2.62 (1.40–4.89)	5.50 (1.76–17.23)	

*Confounders adjustment was performed as in Model 2. Multivariable logistic regression models were used to calculate ORs with a 95% CI. As the number of patients with COPD, liver failure or cirrhosis, stroke, and malignancy is too small in the cohort (especially for the deteriorated subjects), their subgroup analysis could not be performed. ASHD, arteriosclerotic heart disease; CI, confidence interval; HBP, high blood pressure; NLTRP, neutrophil-to-lymphocyte ratio times red cell distribution width-to-platelet ratio; OR, odds ratio; U.C., unable to calculate.*

### Anti-inflammatory Treatments Decrease NLTRP After mTBI on the Mouse Model

**FIGURE 3:**
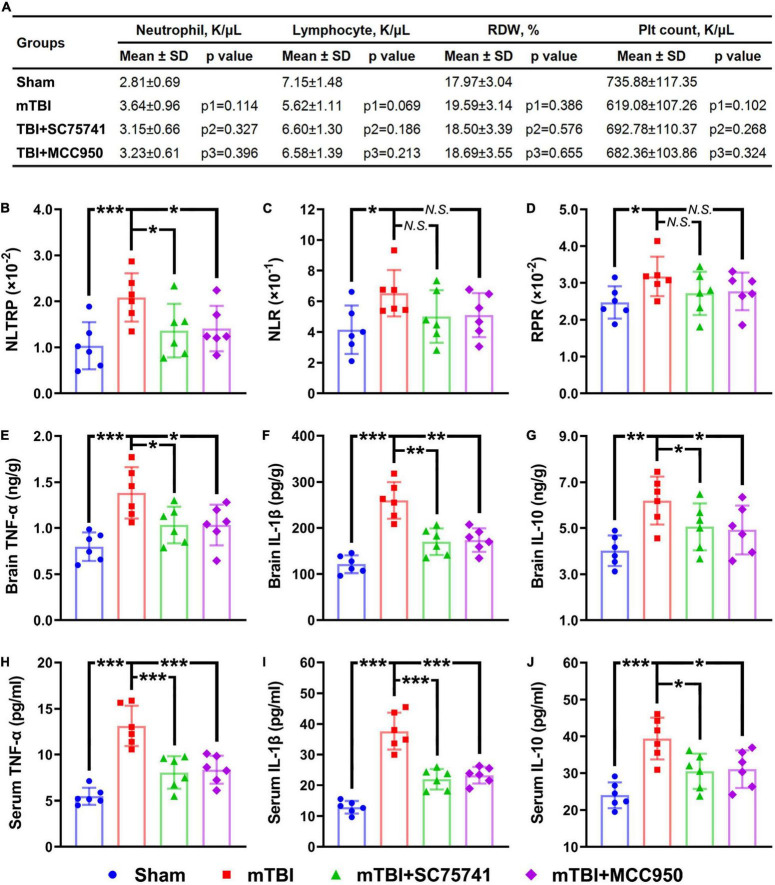
NLTRP changes after mTBI and anti-inflammatory treatments in the mice model. **(A)** Blood neutrophil count, lymphocyte count, RDW, and platelet count of the mTBI mice. **(B–D)** NLTRP, NLR, and RPR levels of the mTBI mice. **(E–G)** The expression levels of inflammatory mediators TNF-α, IL-1β, and IL-10 in the mice brain. **(H–J)** Blood TNF-α, IL-1β, and IL-10 levels of the mTBI mice (n = 6/group). Data are presented as mean ± SD. p1, control vs. mTBI group; p2, mTBI vs. mTBI + SC75741 group; p3, mTBI vs. mTBI + MCC950 group. ***p < 0.001, **p < 0.01, *p < 0.05. mTBI, mild traumatic brain injury; NLR, neutrophil-to-lymphocyte ratio; NS, no significance; RDW, red cell distribution width; RPR, red cell distribution width-to-platelet ratio; NLTRP, neutrophil-to-lymphocyte ratio times red cell distribution width-to-platelet ratio.

*In vivo* experiments on the mTBI mice were designed to further explain the clinical findings. Briefly, the whole blood neutrophil count, lymphocyte count, RDW, and platelet count were all determined at 6 h post-injury, which has been widely reported to be the first time-point of the inflammation peak in mTBI mice (Zhang et al., 2014; Hubbard et al., 2021). We found that the combinatorial indices NLTRP, NLR, and RPR were all increased significantly after mTBI, while no statistical differences in neutrophil count, lymphocyte count, RDW, and platelet count could be observed (Figures 3A–D). In addition, the anti-inflammatory agents SC75741 and MCC950 that inhibited the expression levels of inflammatory mediators (TNF-α, IL-1β, and IL-10) in the brain and serum (Figures 3E–J) could reverse the level change in NLTRP post-injury (Figure 3B). These results suggest that NLTRP is a powerful indicator for inflammation after mTBI, thus a good biomarker for the deterioration risk.

## Discussion

Aging patients with mTBI have a small but clinically important risk of deterioration, giving rise to a wide variety of hospital admission practices in different regions and medical centers. Overutilization of the inpatient ward and intensive care unit for these patients leads to increased healthcare costs, decreased hospitalization resource availability, delays in admitting other critically ill patients, and subsequent emergency department crowding. But underuse of the inpatient ward may lead to poor outcomes due to the delayed recognition of clinical deterioration (Lewis et al., 2020). To solve the problem, cranial CT and MRI have been more and more widely applied in the emergency department in recent years. In addition, many researchers have focused on the early prediction of prognosis in patients with TBI. At present, two highly influential prognostic models, CRASH (Perel et al., 2008) and IMPACT (Steyerberg et al., 2008) have been established. However, both of them were not generated specifically for mTBI. The development populations for both models were weighted toward severe TBI, and patients with mild and moderate TBI were underrepresented (Maas et al., 2017). Therefore, their accuracy in predicting the prognosis of mTBI needs to be further evaluated. From this, we designed this study to explore a routinely available and reliable biomarker to predict the deterioration risk of mTBI and found a novel indicator from CBC analysis, NLTRP. The research findings do not mean NLTRP can replace cranial CT or MRI for making important medical decisions by the emergency doctor, such as whether to admit a patient to the inpatient department. Instead, we recommend using NLTRP as an auxiliary tool combined with clinical and imaging manifestations to evaluate the deterioration risk for patients with mTBI, especially in primary medical institutions without CT or MRI equipment.

There is sufficient laboratory evidence for neuroinflammation and systemic inflammation following mTBI, characterized by level changes of inflammatory markers in the acute phase after brain injury (Visser et al., 2021). However, patients with mTBI are not likely to accept invasive techniques, such as lumbar puncture, to take a cerebrospinal fluid examination. In clinical practice, the inflammatory indicator change that is most likely to draw attention from doctors is the abnormal increase in white blood cell count in circulation, but it does not have any clinical guiding significance for the lack of specificity. Recent studies have suggested that several systemic inflammatory indicators may correlate with the development and outcome of mTBI (Visser et al., 2021). Specifically, NLR has been widely investigated as an available prognostic biomarker and inflammatory indicator for TBI (Sabouri et al., 2020). It represents the degree of secondary brain damage led by neutrophils and their products. Neutrophils are among the first leukocytes whose number increases dramatically in the peripheral blood and enter the central nervous system through the damaged blood–brain barrier soon after TBI. They exacerbate oxidative stress, neurovascular unit damage, and neuronal cell death. However, lymphocytes are important for the repair of damaged brain tissue by releasing growth factors and regulating microglial function (Sabouri et al., 2020). Although higher NLR is correlated with positive CT findings in patients with mTBI (Alexiou et al., 2020), it has a relatively low discriminative value to predict secondary neurological impairment (Le Bail et al., 2021). Additionally, in two independent clinical cohorts of mild, moderate, and severe TBI, RPR is a desirable prognostic biomarker and an indicator for the development of inflammatory response (Ge et al., 2021; Wang et al., 2021). In this regard, RDW characterizes the heterogeneity in the size of erythrocytes and is widely utilized as an indicator to differentiate types of anemia. Under inflammation status, the pro-inflammatory cytokines could inhibit erythrocyte maturation and accelerate the release of larger reticulocytes into blood circulation, thus increasing RDW (Sun et al., 2016). Besides, along with the well-known roles in hemostasis, platelets are also crucial regulators in local and systemic inflammation. Increased platelet destruction and consumption could be caused by infectious and inflammatory damage to megakaryocytes (Levi, 2016). Nevertheless, in the pre-analysis for the mTBI cohort of this study, RPR was found to have a poor relationship with the deterioration risk of mTBI. Therefore, we selected NLTRP to be the potential biomarker for further research. As it is the product of NLR and RPR, it could amplify the prediction value of NLR and RPR in theory.

Our findings from the clinical cohort provided solid evidence for recognizing the deterioration risk of mTBI using NLTRP. Although the exact mechanism underlying the deterioration after mTBI with elevated NLTRP remains unclear, it may be partially attributed to the development of neural and systemic inflammatory responses following brain injury. Consequently, we further conducted *in vivo* experiments to explain possible mechanisms. As an upstream switch of inflammation, NF-κB signaling exerts critical effects on regulating the development of neuroinflammation in mTBI (Ghadiri et al., 2020). Besides, the NLRP3 inflammasome could trigger the inflammatory cascade (pyroptosis) in the injured brain after mTBI (O’Brien et al., 2020). In this study, we used specific NF-κB and NLRP3 inhibitors to observe the level changes in NLTRP, NLR, and RPR under the condition of inflammatory suppression. NLTRP, NLR, and RPR were all observed to be increased after mTBI, and NLTRP was also decreased, notably after the above treatments. These results confirmed the conserved response to TBI between species and proved the hypothesis that increased NLTRP was a manifestation of the inflammatory response after mTBI.

Notably, inflammation is one of the main pathological mechanisms leading to secondary injury after an acute insult to the brain, not only for neurotrauma but also for ischemic and hemorrhagic injury. Inflammatory biomarkers, including NLR and the systemic inflammatory response index, have been proved to be independently associated with the deterioration of stroke patients, thus have great potential to be available for clinical practice due to their easy accessibility and cost-effectiveness (Lattanzi et al., 2017, 2021; Świtońska et al., 2020). It further suggests the wide availability of inflammatory indicators, such as NLTRP, in the prognosis evaluation for various neurological diseases.

Several limitations, especially the retrospective nature of the design, should be considered when interpreting the results of this study. On the one hand, patients’ information for cranial CT scanning, Abbreviated Injury Scale—Injury Severity Score at admission, and Glasgow Outcome Scale—Extended were not collected in MIMIC-IV and eICU-CRD, which led to the absence of these data in the cohort. Although similar research using the GCS sub-score from the databases has reported a better machine-learning prognosis model, it may be difficult for clinical application as the model includes up to 20 features (Palepu et al., 2021). On the other hand, an external validation dataset needs to be built in the future to further verify the research findings. From this, a multicenter prospective study is being organized by our research group aiming at promoting the clinical application of NLTRP for patients with mTBI.

## Conclusion

NLTRP is a promising indicator for the deterioration risk of patients with mTBI. Its level change after brain injury is attributed to the development of neural and systemic inflammatory responses, which further emphasizes the importance of controlling inflammation in clinical treatment. Due to its low cost, easy availability, and high reproducibility, NLTRP can be regarded as a reliable clinical tool at all levels of the medical system.

## Data Availability Statement

The original contributions presented in the study are included in the article, further inquiries can be directed to the corresponding author.

## Ethics Statement

XG completed the National Institutes of Health’s web-based course Protecting Human Research Participants (certification no: 36320014) to access the databases. Written informed consent for participation was not required for this study in accordance with the national legislation and the institutional requirements. The animal study was reviewed and approved by Tianjin Medical University Animal Care and Use Committee.

## Author Contributions

XG and PL contributed to the conception and design of the study. XG and LZ developed the method and performed data extraction and statistical analysis. LZ conducted laboratory examinations. ML conducted *in vivo* experiments. FC, YL, and JZ provided methodological and technical support. XG wrote the manuscript. PL reviewed the manuscript. All authors contributed to manuscript revision, read, and approved the submitted version.

## Conflict of Interest

The authors declare that the research was conducted in the absence of any commercial or financial relationships that could be construed as a potential conflict of interest.

## Publisher’s Note

All claims expressed in this article are solely those of the authors and do not necessarily represent those of their affiliated organizations, or those of the publisher, the editors and the reviewers. Any product that may be evaluated in this article, or claim that may be made by its manufacturer, is not guaranteed or endorsed by the publisher.

## Abbreviations

ASHD: arteriosclerotic heart disease
AUC: area under ROC curve
CBC: complete blood count
CCI: controlled cortical impact
CI: confidence interval
COPD: chronic obstructive pulmonary disease
CT: computed tomography
DCA: decision curve analysis
eICU-CRD: eICU collaborative research database
GCS: glasgow coma scale
HBP: high blood pressure
IDI: integrated discrimination improvement
MeDICS: MIMIC-III and eICU database integration cases study
MIMIC-III: multiparameter intelligent monitoring in intensive Care III
mTBI: mild traumatic brain injury
NLR: neutrophil-to-lymphocyte ratio
NLTRP: neutrophil-to-lymphocyte ratio times RDW-to-platelet ratio
OR: odds ratio
RDW: red cell distribution width
ROC: receiver-operating characteristic
RPR: RDW-to-platelet ratio.
